# Cannabinoids and healthy ageing: the potential for extending healthspan and lifespan in preclinical models with an emphasis on *Caenorhabditis elegans*

**DOI:** 10.1007/s11357-024-01162-8

**Published:** 2024-05-02

**Authors:** Zhizhen Wang, Jonathon C. Arnold

**Affiliations:** 1 Lambert Initiative for Cannabinoid Therapeutics, Brain and Mind Centre, The University of Sydney, Sydney, NSW Australia; 2 Discipline of Pharmacology, Sydney Pharmacy School, Faculty of Medicine and Health, The University of Sydney, Sydney, NSW Australia

**Keywords:** Cannabidiol,, Lifespan,, *C. elegans*,, Health span,, Endocannabinoid system

## Abstract

There is a significant global upsurge in the number and proportion of older persons in the population. With this comes an increasing prevalence of age-related conditions which pose a major challenge to healthcare systems. The development of anti-ageing treatments may help meet this challenge by targeting the ageing process which is a common denominator to many health problems. Cannabis-like compounds (cannabinoids) are reported to improve quality of life and general well-being in human trials, and there is increasing preclinical research highlighting that they have anti-ageing activity. Moreover, preclinical evidence suggests that endogenous cannabinoids regulate ageing processes. Here, we review the anti-ageing effects of the cannabinoids in various model systems, including the most extensively studied nematode model, *Caenorhabditis elegans*. These studies highlight that the cannabinoids lengthen healthspan and lifespan, with emerging evidence that they may also hinder the development of cellular senescence. The non-psychoactive cannabinoid cannabidiol (CBD) shows particular promise, with mechanistic studies demonstrating it may work through autophagy induction and activation of antioxidative systems. Furthermore, CBD improves healthspan parameters such as diminishing age-related behavioural dysfunction in models of both healthy and accelerated ageing. Translation into mammalian systems provides an important next step. Moreover, looking beyond CBD, future studies could probe the multitude of other cannabis constituents for their anti-ageing activity.

## Introduction

Ageing is a complex biological process that involves the gradual deterioration of cellular and molecular processes over time. This process is influenced by a variety of factors, including genetics, lifestyle and environmental factors [1]. During ageing, our bodies gradually become less efficient at repairing damage and defending against disease [2], which can result in an overall decline in health and an increased risk of chronic conditions such as cardiovascular disease [3], diabetes [4] and cancer [5]. Age-related changes can affect almost every system in the body, including the immune system [6], the cardiovascular system [7] and the nervous system [8]. These changes can result in a variety of functional impairments, such as decreased mobility, impaired cognition and reduced sensory function, that can significantly impact the quality of life and independence [9, 10].

As the global population continues to age, there is increasing interest in identifying strategies that can promote healthy ageing and extend lifespan. Because of the challenges brought by an ageing society, the United Nations declared 2021–2030 the *UN Decade in Healthy Ageing* and has called for society to increase efforts to develop innovative means to improve both the healthspan and lifespan of the human race. Basic research can contribute to this effort via the discovery of chemical agents that improve healthy ageing. The development of anti-ageing therapies provides a paradigm shift in treating disease. As many health conditions are age-related, diminishing age-related decline might provide a common means to prevent a spectrum of diseases and comorbidities that plague the community.

The legalisation of medicinal cannabis around the world has prompted the question of whether cannabinoids (cannabis-like drugs) might promote longevity and mitigate age-related decline. There is an emerging picture from clinical trials on medicinal cannabis products that they improve the general quality of life and well-being of patients [11]. However, controlled studies are needed to specifically address whether cannabinoids can extend the healthspan (the number of years having functional health) and lifespan. In this review, we explore the potential benefits of cannabinoids to healthy ageing and ageing-related disease by examining whether the endogenous cannabinoid (endocannabinoid) system and exogenous cannabinoids extend healthspan and lifespan in preclinical models. While beyond the scope of the present review, it is important to acknowledge that cannabinoid exposure may negatively impact early development. The interested reader is encouraged to see the following excellent reviews on the developmental impacts and toxicity of cannabinoid exposure at different ages (e.g. prenatal, adolescent or adult) [12–15].

## Background on the cannabinoids

Cannabinoids are chemical compounds that are found in the *Cannabis* plant [16]. Over 100 plant cannabinoids (phytocannabinoids) have been elucidated, with the most characterised being the main psychoactive constituent, Δ^9^-tetrahydrocannabinol (THC), and the non-intoxicating constituent cannabidiol (CBD). Whilst being structural isomers, these major cannabinoids have a distinct pharmacology and potential therapeutic applications [17–19]. At present, THC-containing products are registered treatments for chemotherapy-induced nausea and vomiting, and spasticity associated with multiple sclerosis, whereas CBD is a registered drug to treat intractable epilepsies such as Dravet syndrome and Lennox-Gastaut syndrome [20, 21]. There is currently intensive research activity exploring additional therapeutic applications for the cannabinoids. These include studies on molecules such as cannabidiolic acid, cannabigerolic acid, cannabigerol (CBG), cannabichromene (CBC) and cannabinol (CBN), among others [22–26].

**Fig. 1:**
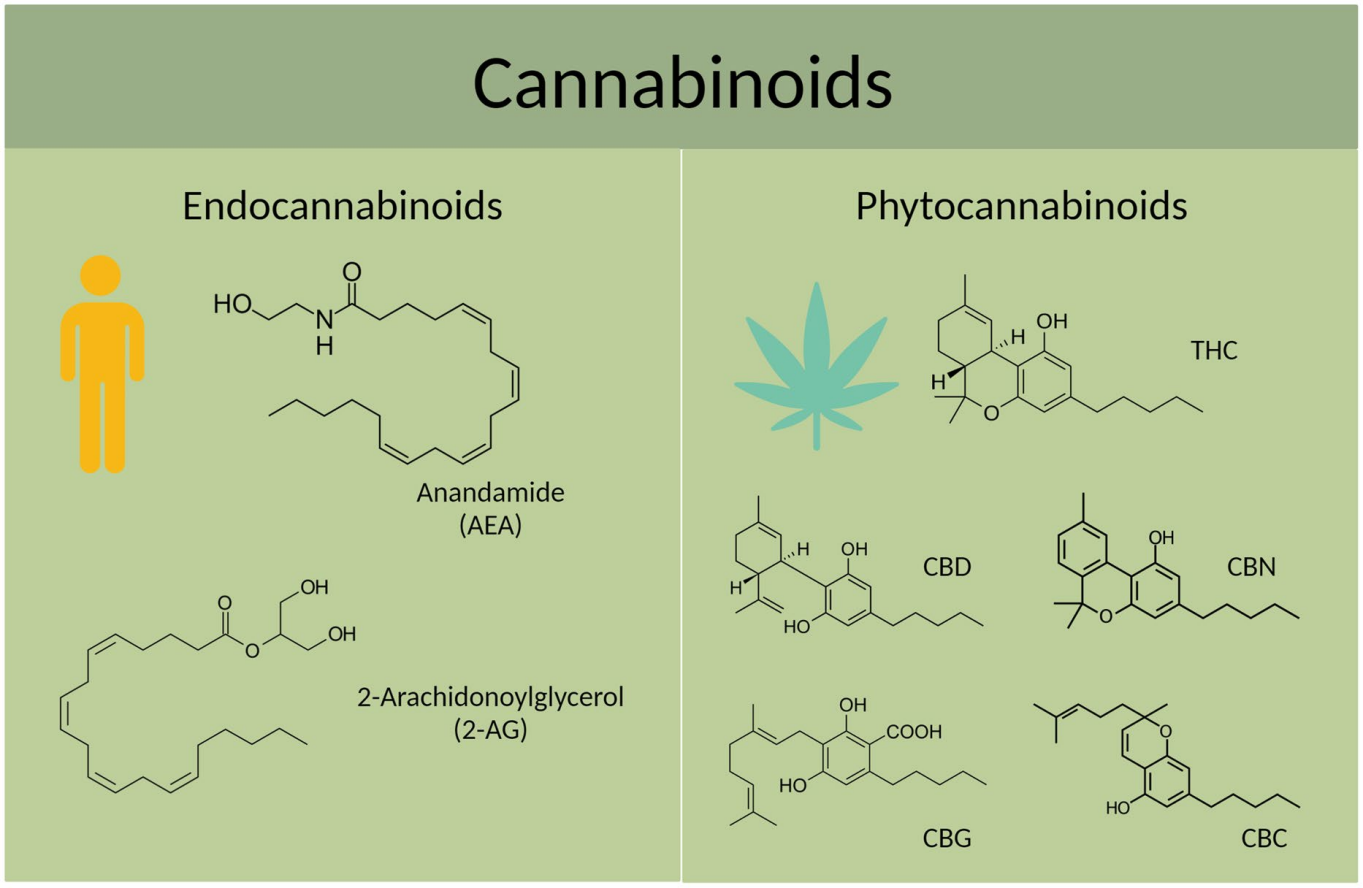
Chemical structures of endocannabinoids (produced naturally by the body) and major phytocannabinoids (produced by plants). THC, Δ^9^-tetrahydrocannabinol; CBD, cannabidiol; CBN, cannabinol; CBG, cannabigerol; CBC, cannabichromene

One of the main modes of action of the phytocannabinoids THC and CBD is to modulate the body’s endocannabinoid system (ECS) (Fig. 1) [27]. This is a complex signalling system that plays a role in regulating various physiological processes such as mood, appetite, pain sensation, sleep and immune function [28–31]. The ECS is composed of a network of receptors, endocannabinoids and enzymes that work together to maintain homeostasis. The two primary receptors of the ECS are cannabinoid receptor type-1 (CB1R) and cannabinoid receptor type-2 (CB2R), which are found throughout the body, including in the brain, immune system and peripheral tissues [32, 33]. Two of the most extensively studied endogenous ligands are the endocannabinoids anandamide (AEA) and 2-arachidonoylglycerol (2-AG), as well as the enzymes responsible for the metabolism of endocannabinoids, including fatty acid amide hydrolase (FAAH) and monoacylglycerol lipase (MAGL). THC directly activates cannabinoid receptors CB1R and CB2R to produce its pharmacological actions, whereas CBD is thought to be an indirect agonist that boosts endogenous concentrations of the endocannabinoids like AEA via inhibition of fatty acid-binding proteins (FABPs) [34], such as FABP5 and FABP7 [35]. FABP5 plays a key role in retrograde endocannabinoid signaling and acts as a carrier of endocannabinoids at synapses in the central nervous system [36]. It should also be recognised that CBD has a multitude of pharmacological actions beyond the ECS, including antagonism of G protein-coupled receptor 55 (GPR55), activation of transient receptor potential vanilloid-1 (TRPV1) channels and positive allosteric modulation of γ-aminobutyric acid sub-type A (GABA_A_) receptors [37–42].

## Ageing and the ECS

**Fig. 2:**
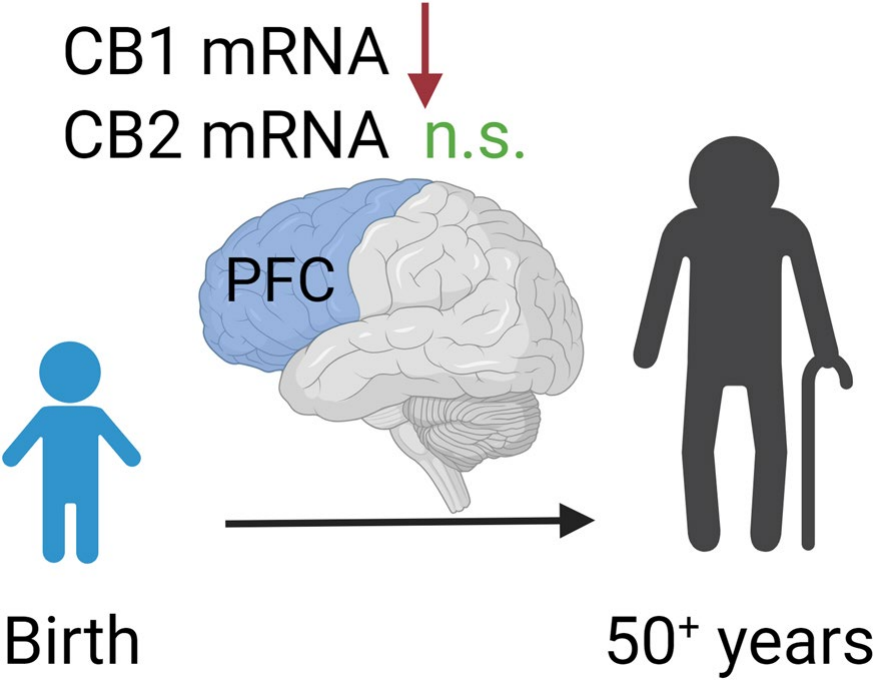
Ageing decreased mRNA expression of cannabinoid CB1R but not CB2R in the PFC of humans with ages ranging from birth to 50 years. PFC, the prefrontal cortex; CB1R, cannabinoid CB1 receptors; CB2R, cannabinoid CB2 receptors; n.s., not significant

Several changes occur in the ECS during ageing that may have health implications. In the human brain, CB1R mRNA expression gradually decreased with age in the prefrontal cortex (PFC) (Fig. 2) [43]. In addition, aged rats displayed increased CB1R protein expression in the entorhinal and temporal cortices but decreased expression in the postrhinal cortex compared to young rats [44]. Further, aged female mice showed increased CB1R mRNA expression in subregions of the cortex compared to young mice [45]. However, aged mice exhibited decreased CB1R mRNA expression in the lateral septal area compared to younger animals [45].

Age-related changes in the brain ECS are accompanied by decrements in physiological function. Genetic deletion of CB1R in mice led to an earlier decline in cognitive and memory function specific to both reward and aversion-driven learning [46–48]. Young mice lacking CB1R exhibited comparable or even improved learning and memory performance compared to age-matched wild-type (WT) mice in various learning and memory tasks. In contrast, mature mice lacking CB1R performed significantly worse than age-matched WT mice in the same tasks. Additionally, the rapid decline in cognitive functions observed in mature CB1R-deficient mice was accompanied by a loss of neurons in the CA1 and CA3 regions of the hippocampus [46]. A noteworthy finding suggests that a decrease in CB1 signaling specifically in the limbic forebrain contributed to age-related decline in appetite for food and alcohol [49].

In addition to the involvement of CB1R signaling in the ageing process, CB2R may also play a role. CB2R is abundantly expressed in immune cells, including the brain’s immune cells, the microglia. CB2R is upregulated during inflammation and could contribute to inflammaging, the chronic, low-grade inflammation that occurs during ageing. Consistent with this notion, unlike WT mice that display an age-related decline in social memory, CB2R knockout mice were resistant to such a decline and even showed some improvement [50]. This effect was accompanied by various molecular and morphological changes in the microglia of the CB2R knockout mice. Interestingly, another study revealed that β-caryophyllene, a natural agonist of CB2R, reversed age-related impairments in working memory and reduced circulating inflammatory cytokine concentrations in aged mice [51].

It is uncertain whether tissue concentrations of the endocannabinoids are altered during the ageing process. Concentrations of AEA and 2-AG did not exhibit age-dependent changes in various brain regions such as the hypothalamus, limbic forebrain, amygdala and cerebellum [49]. A recent study investigated changes in the ECS during ageing in the mouse brain [47]. The ECS was most impacted in middle-aged mice compared to young adult mice. Specifically, middle-aged mice displayed a prominent decrease in 2-AG in the hippocampus, whereas AEA was decreased in other regions. There is evidence that ageing influences the expression and activity of enzymes that synthesize and degrade endocannabinoids. For example, aged animals exhibited reduced expression of the 2-AG synthesising enzyme DAGL [47], but increased expression of the AEA synthesising enzyme *N*-acyl phosphatidyl ethanolamide-phospholipase D (NAPE-PLD) compared to younger mice [52]. Another study reported age-related alterations in ECS components in female reproductive organs [53]. In conclusion, the ECS undergoes various changes during the ageing process and appears functionally implicated in the protection against age-related physiological decline.

## Model systems for studying ageing

Various model systems, such as fruit fly (*Drosophila*) [54, 55], nematodes (*Caenorhabditis elegans*) [56] and zebrafish (*Danio rerio*) [57], have offered valuable insights into the ageing process. These model organisms are particularly advantageous due to their relatively short lifespans, which have enabled the elucidation of the molecular, genetic and physiological factors that influence ageing and age-related diseases [58–60]. Rodents are also employed as an ageing model because of their genetic similarity to humans, and genetic rodent models of accelerated ageing have been developed. However, while important, rodent ageing studies are time-consuming and of much lower throughput in the discovery phase of ageing research [61]. Lifespan is the number of years someone lives from birth until death, while healthspan refers to the period of life spent in good health, free from age-related diseases and disabilities [62]. Model systems used to study ageing incorporate both measurements of lifespan and healthspan, as there is no point in increasing longevity without a corresponding improvement in overall health and well-being.

**Fig. 3:**
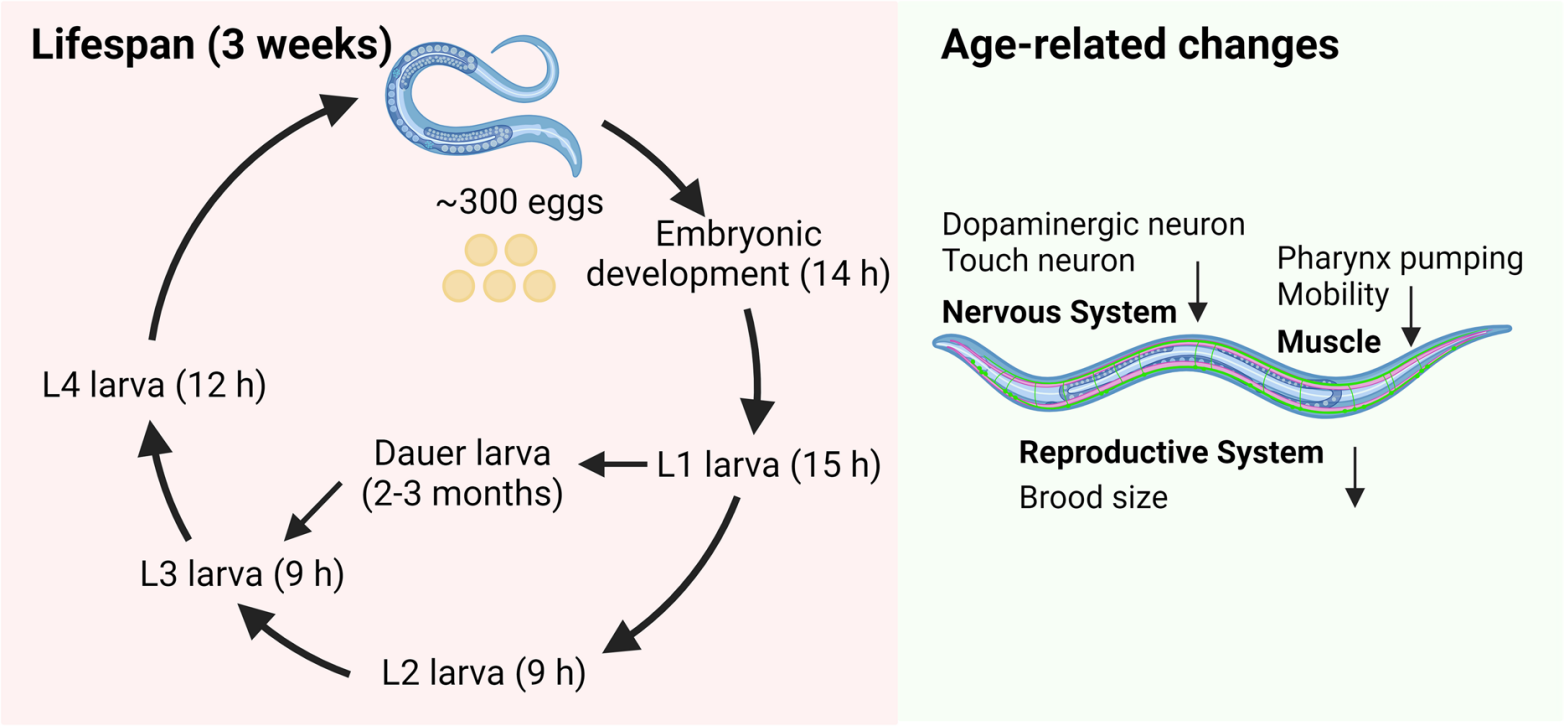
The life cycle of *C. elegans* at 20 °C and age-related changes that are measured in geroscience research. Under normal laboratory conditions, an adult hermaphrodite produces roughly 300 eggs that hatch after 14 h. In favourable conditions, *C. elegans* goes through four larval stages (L1 to L4) to the adult in 3–5 days. In unfavourable conditions (overcrowding/low food/high temperature), the L1 larva may choose the dauer larva, which can live for several months. The overall lifespan of *C. elegans* is 2–3 weeks. Age-related changes in the nervous system, muscle and reproductive system

Here, we provide a more detailed background on the nematode *C. elegans,* which has been the most widely used model when studying the effects of cannabinoids on lifespan and healthspan. More generally, the *C.elegans* model is used in ageing research due to the ability to rapidly discern age-related physiological decline over their short lifespan (~ 3 weeks). *C. elegans* is a free-living nematode with a small size (1 mm in length) and is easy to maintain in laboratory settings. *C. elegans* can develop from eggs to adults in about 3 days. The growth of *C. elegans* contains four larval stages (L1–L4) and an adult stage (Fig. 3). These convenient features make it particularly suitable as a model system for studies on ageing, age-related diseases and mechanisms of longevity.

**Fig. 4:**
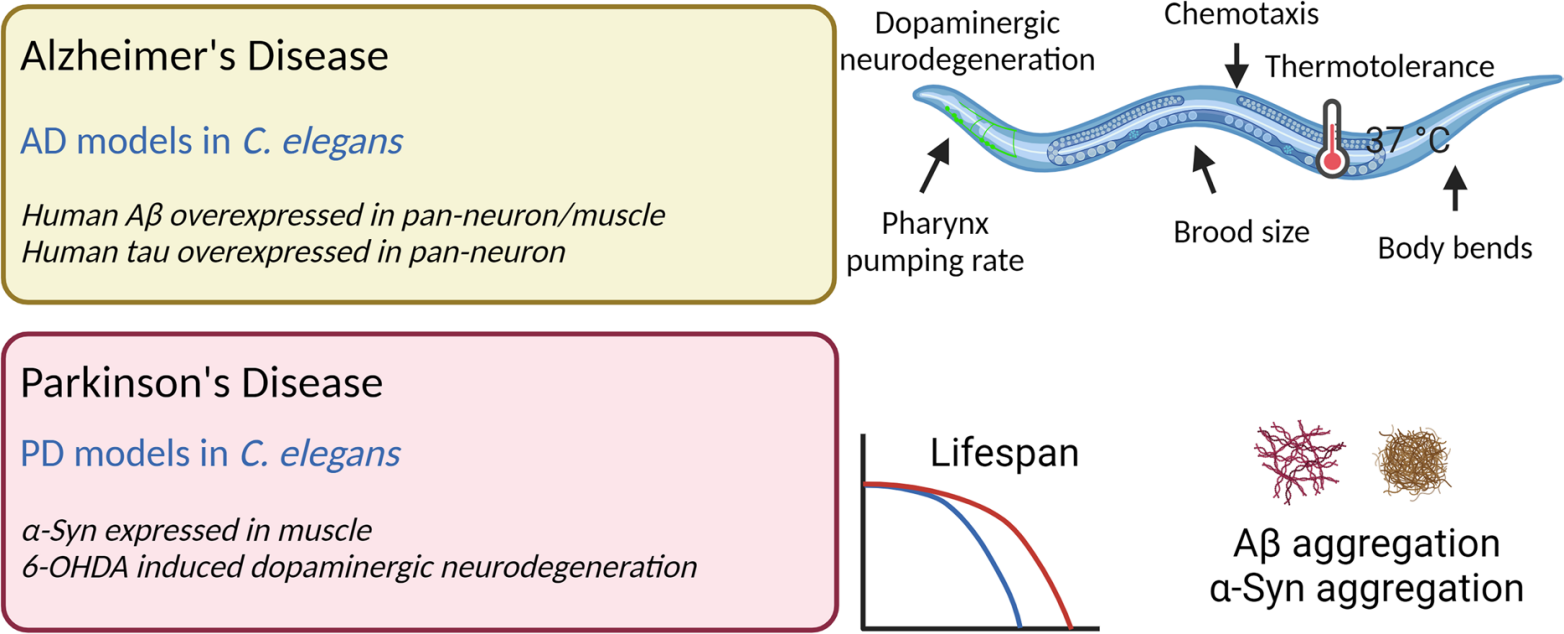
Neurodegenerative disease models in *C. elegans* used to study cannabinoid effects on healthspan and lifespan. Created with www.biorender.com

Age-related changes are evident in various tissues of *C. elegans*, including the cuticle (skin) and the reproductive and nervous systems [63] (Fig. 3). The ageing intestine of *C. elegans* displays deterioration of intestinal microvilli [64]. Fertility significantly declines with age, and the reproductive system deteriorates due to factors such as sperm shortage and reduced oocyte size and quality [65, 66]. During the ageing process, unmated *C. elegans* experience a decline in brood size [67, 68]. Age-related morphological changes in neurons have been extensively documented in *C. elegans*. Although earlier reports suggested no neuron loss or axon deficits in ageing worms, recent studies have identified delicate yet consistent age-dependent alterations in the organism’s neurons [69]. In adult hermaphrodites, dopaminergic and touch receptor neurons, exhibit age-related changes such as gradual beading, blebbing and branching [70]. Thus, using *C. elegans* models provides a clear benefit, as it allows for the study of age-related neurodegeneration and the discovery of potential therapeutic compounds for ageing and age-related diseases such as Alzheimer’s disease (AD) and Parkinson’s disease (PD) (Fig. 4).

Phenotypic testing in nematodes has led to the discovery of anti-ageing compounds, and some of these compounds are already in clinical use. For example, metformin, an anti-diabetic drug, extends lifespan and healthspan in nematodes and in mouse models of ageing [71, 72]. Beyond its anti-diabetic effects, population studies have found that metformin use decreased the risk of developing cancer [73], cognitive impairment and dementia [74] and cardiovascular disease [75]. Metformin has also been reported to reduce the risk of spontaneous abortions and obstetric complications [72]. There is increasing evidence that metformin improves general well-being beyond its diabetic mode of action and through classic anti-ageing pathways [72]. This has prompted calls for evidence on whether metformin has health benefits in non-diabetic patients. In one smaller scale, double-blind placebo-controlled study in healthy elderly participants, metformin was shown to impact non-metabolic pathways linked to ageing [76]. Providing a roadmap for future clinical studies, a larger scale, multi-site trial called TAME (targeting ageing with metformin) aims to enrol 3000 people in the 65–79 age range with a primary endpoint of time until age-related morbidity (e.g. coronary heart disease, cancer, dementia) and mortality [72]. Three other trials (phase 1 and 2) are also underway looking at whether metformin improves physical performance and reduces frailty [72].

## Anti-ageing effects of endogenous cannabinoids in *C. elegans*

*C.elegans* contain an ancestral endocannabinoid system including the endocannabinoid signalling lipids AEA and 2-AG [77]. The AEA biosynthetic enzyme NAPE-PLD [78] has two functional orthologs in *C. elegans*, namely *nape-1* and *nape-2*, which have overlapping expression in the pharynx and brain interneurons [79]. Overexpression of *nape-1* and *nape-2* in worms produced distinct growth phenotypes at different ambient temperatures [80]. Overexpression of *nape-1* reduced lifespan only at 25 °C, whereas overexpression of *nape-2* extended lifespan at 15 °C.

The nematodes have an ortholog of the AEA catabolic enzyme FAAH, known as *faah-1*. *Faah-1* is predominantly expressed in the pharynx where it is co-expressed with *nape-2* [79]. Reducing the levels of *faah-1* using RNA interference (RNAi) in worms increased concentrations of *N*-acyl ethanolamines (NAEs), including AEA. Dietary restriction, known to diminish NAE concentrations, extended the lifespan of worms [81, 82]. Further, the overexpression of *faah-1* in the pharynx also increased lifespan [79]. The ortholog of the 2-AG biosynthetic enzymes DAGLα/DAGLβ has an orthologue in *C. elegans* named *dagl-1*. Overexpression of *dagl-1* in nematodes increased lifespan, whereas the genetic downregulation of *dagl-1* decreased lifespan and resistance to paraquat-induced oxidative stress [83]. It is worth noting that *C. elegans* lacks an ortholog for the 2-AG-metabolising enzyme MAGL. Interestingly, a recent study reported that the MAGL inhibitor JZL184 extended the lifespan of *C. elegans* [84]. Notably, activity-based protein profiling determined *faah-4* (ortholog of FAAH in *C. elegans*) as the major target of JZL184 in this study. Collectively, these findings indicate that *C. elegans* may have a complete and functional endocannabinoid signalling pathway, similar to mammals, which regulates ageing and lifespan.

The ECS in *C. elegans* also subserves locomotion, nociception, reproduction, feeding and axon regeneration. AEA was reported to bind the G-protein-coupled receptors (GPCRs) NPR-19 and NPR-32 in *C. elegans*, and the activation of these receptors suppressed axon regeneration [85]. Although the nematode NPR-19 ortholog has only 23% similarity to human CB1R, an analysis revealed that the crucial amino acids responsible for AEA binding seem to be preserved across species [86, 87]. Furthermore, 2-AG or AEA activated NPR-19 to inhibit nociception and feeding. The endocannabinoids also work through other receptors such as OCTR-1 (α_2A_-adrenergic-like octopamine receptor) and SER-4 (5-HT_1A_-like serotonin receptor) to influence nociception and locomotion [87]. Studies found that 2-AG acted as an endogenous modulator of TRPV signal transduction, influencing sterol mobilisation via the IGF-1 signalling pathway [88] and stimulating cholesterol trafficking [89] in nematodes. AEA has a bidirectional effect on feeding in *C. elegans*, which is mediated by NPR-19 [90]. Interestingly, the effect of genetic deletion of NPR-19 on food preference was rescued by either introducing overexpression of NPR-19 or overexpression of the human CB1 receptor. This reinforces that there is a high degree of conservation of nematode and human endocannabinoid systems. Furthermore, functional conservation of the ECS for regulating food preferences was linked to the modulation of AWC chemosensory neurons [90].

## Exogenous plant cannabinoid effects on lifespan in *C. elegans*

There is accumulating research showing that plant cannabinoids extend lifespan and healthspan in nematodes. Much of the research has focussed on the non-psychoactive cannabinoid constituent CBD, which is being sold as an over-the-counter wellness product in many countries [18, 91]. Recent evidence shows that CBD increased longevity in *C.elegans* in both wild-type (“healthy”) nematodes and also in nematode models of neurodegeneration (Table 1). We will now review these studies in greater detail by examining the magnitude of the effect of CBD in *C.elegans* models, as well as examining potential anti-ageing mechanisms in this species.

**Table 1:** Lifespan studies of CBD treatment in *C. elegans*

Strains	Genotype	Dose	Main effects	Pathway/regulator	Refs
N2	Wild type	10 µM, 40 µM and 100 µM CBD	↑ Mean lifespan 14.8% (10 µM), 18.3% (40 µM) and 12.2% (100 µM)	N/A	[92]
N2	Wild type	100 µM CBD	↑ Lifespan at days 7,21, 25, 29 and 33	Glyoxalase pathway	[93]
VC343	*glod-4(gk189)*	100 µM CBD	↑ Lifespan in glod-4 knockout strain at days 13, 17, 21 and 29	Neural Glyoxalase Pathway	
VH725	hdEx231 [C16C10.10::GFP + pha-1( +)]. Ubiquitous expression of glyoxalase-1::GFP	100 µM CBD	↑ Lifespan in overexpressing glod-4 model at day 25, 29, 33 and 37	Neural Glyoxalase Pathway	
GRU102	gnaIs2 [myo-2p::YFP + unc-119p::Abeta1-42]. Pan-neuronal amyloid beta1-42 expression	100 µM CBD	↑ Lifespan in AD model at days 17, 21, 25 and 29	Lowered Aβ expression	
N2	Wild type	1 µM, 5 µM and 10 µM CBD	↑ Mean lifespan 23.1% (1 µM), 7.7% (5 µM) and 10.9% (10 µM)	Autophagy pathway	[94]
N2	*vps-34* RNAi	1 µM CBD	CBD treatment failed to extend lifespan in *vps-34* RNAi group	Autophagy pathway	
N2	*bec-1* RNAi	1 µM CBD	CBD treatment failed to extend lifespan in *bec-1* RNAi group	Autophagy pathway	
N2	*sqst-1* RNAi	1 µM CBD	CBD failed to extend lifespan in the *sqst-1* RNAi group	Autophagy pathway	
N2	*sir-2.1* RNAi	1 µM CBD	CBD treatment did not affect the shortened lifespan of *sir-2.1*	SIRT1/AMPK pathway	
RB754	aak-2(ok524)	1 µM CBD	CBD treatment did not affect the shortened lifespan of *aak-2* mutant group	SIRT1/AMPK pathway	
CL2122	dvIs15 [(pPD30.38) unc-54(vector) + (pCL26) mtl-2::GFP]	5 µM CBD	↑ Mean lifespan 40% in control strain for AD	N/A	[95]
CL2355	dvIs50 [pCL45 (snb-1::Abeta 1–42::3′ UTR(long) + mtl-2::GFP]	5 µM CBD	↑ Mean lifespan 25.6% in AD model	N/A	
BZ555	egIs1 [dat-1p::GFP]	25 µM, 50 µM and 100 µM CBD	↑ Mean lifespan 11.5% (25 µM), 23.1% (50 µM) and 28.8% (100 µM) BZ555 pretreated with 6-OHDA	Antioxidative enzymes	[96]
N2	Wild type	100 µM CBD	CBD and CBD derivatives prolonged worm survival in the presence of 200 µM juglone	N/A	[97]
NL5901	pkIs2386 [unc-54p::alphasynuclein::YFP + unc-119( +)]	0–50 µM CBDV	CBDV significantly prolonged the lifespan of PD model worms	DAF-16 pathway	[98]

The first lifelong “toxicity” study of CBD in N2 (Bristol wild-type strain) *C. elegans* was published in 2021 [92]. Whole-life treatment with CBD (10–100 µM) increased lifespan, and a maximum lifespan extension of 18% was observed at 40 µM. CBD also increased thermotolerance as it protected against temperature-induced mortality. Another study in the N2 strain showed 100 µM CBD increased survival at various time points of daily exposure [93]. In addition, CBD also extended lifespan extension at lower concentrations (1 and 5 µM) in the N2 strain [94]. Overall, these results are very promising when you consider that metformin increased mean lifespan by 40% at a much higher concentration (50 mM) in nematodes [71, 72]. Results are also comparable to thioflavin T, which is another very promising anti-ageing compound that has robust lifespan-extending effects in *C. elegans* (25% increase in lifespan at 50 µM) [99]. The ability of CBD to extend the lifespan in *C.elegans* has now been replicated across 3 independent laboratories. Moreover, the effects of CBD were robust across different developmental treatment regimens; positive studies have administered CBD from the egg stage, but also from early adulthood [92–94].

Ageing is the leading risk factor for neurodegenerative diseases, such as AD and PD [100], and the anti-ageing effects of CBD have been observed in worm models of neurodegeneration (Table 1 and Fig. 4). CBD 5 µM increased the lifespan in both the control strain and in an amyloid β (Aβ) overexpression strain of AD by 40% and 25.6% respectively [95]. Dopaminergic neuron degeneration is often observed in AD, and is a contributing factor to cognitive impairment during the early stages of the disease [101, 102]. It was shown that CBD 5 µM reduced Aβ-induced degeneration of dopaminergic neurons [95].

To study PD in *C. elegans*, neurotoxin 6-hydroxydopamine (6-OHDA) models are used which model the selective degeneration of dopaminergic neurons observed in PD patients. CBD at 25, 50 and 100 µM increased lifespan by 18.3%, 32.7% and 45.1% respectively in a concentration-dependent manner in the 6-OHDA dopamine-depletion model of PD [96]. A similar lifespan extension was reported for CBD in the BZ555 strain treated with 6-OHDA (the BZ555 strain is used to visualise dopamine neurons with a GFP tag on the dat-1 promoter) [96]. CBD partially recovered the loss of dopaminergic neurons in 6-OHDA-treated BZ555 worms, as well as food-sensing behaviour deficits [96]. Cannabidivarin (CBDV), is a structural analog of CBD, and showed longevity benefits in the α-synuclein aggregation PD model of *C. elegans* at 10 and 50 µM concentrations [98]. Moreover, CBDV reduced 6-OHDA-induced reductions in the soma size of dopamine neurons and deficits in body bends’ behaviour in BZ555 nematodes [98].

## Mechanisms for the anti-ageing effects of cannabinoids on lifespan in *C. elegans*

**Fig. 5:**
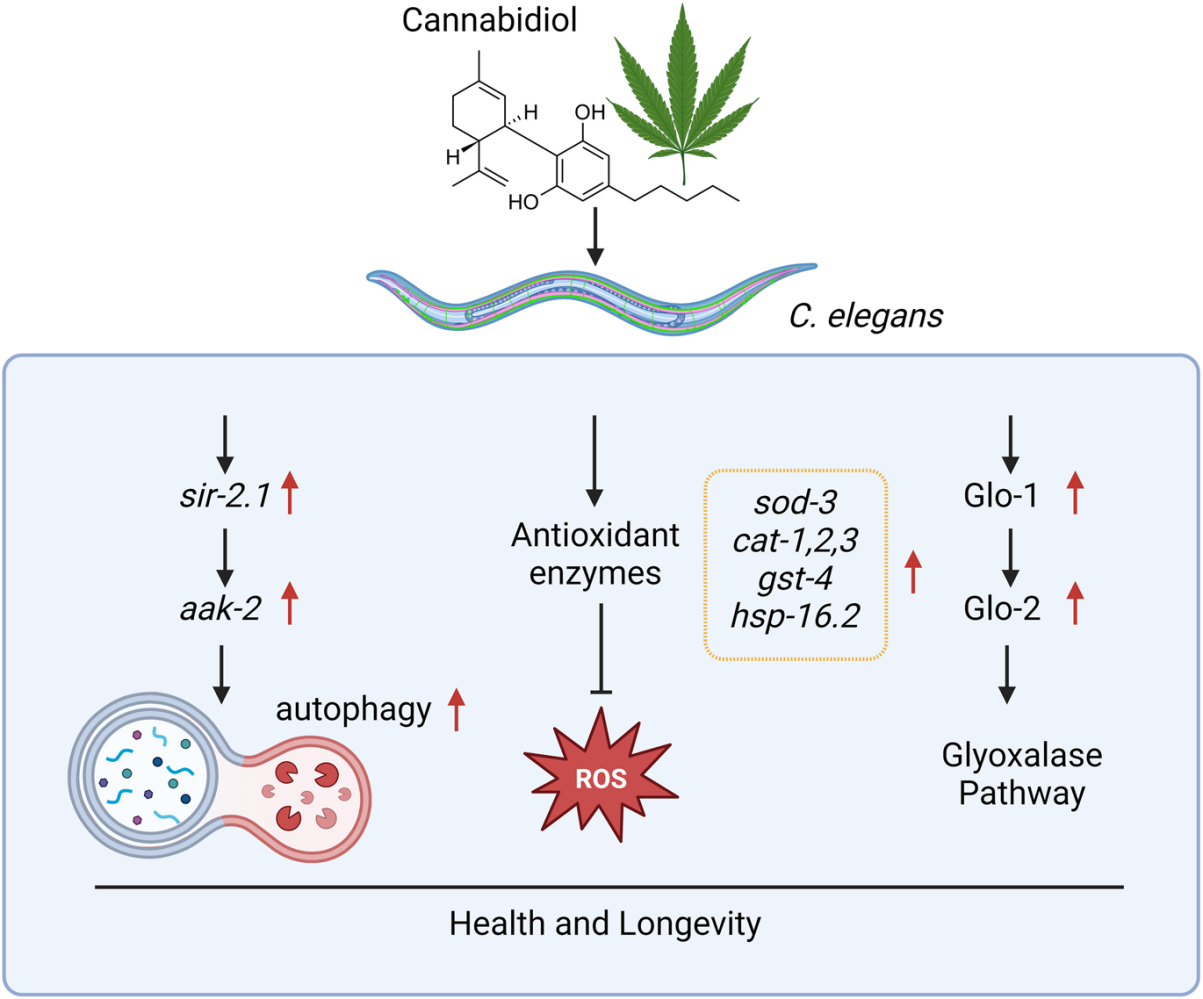
A summary of the known anti-ageing mechanisms of CBD in *C. elegans*. Created with www.biorender.com

Anti-ageing mechanisms of CBD have been elucidated, with nematode research showing that CBD induces autophagy and reduces oxidative stress (Table 1, Fig. 5). Autophagy is a cellular process that removes impaired cellular organelles and debris. Impaired autophagy promotes ageing, whereas increased autophagy has anti-ageing effects [103–105]. Most autophagy genes have orthologs in worms as the process is highly conserved across species [106]. Studies have examined the effects of autophagy genes on CBD-induced lifespan extension using RNAi to knockdown specific genes. Conditional knockdown of *bec-1*, *vps-34* and *sqst-1* abolished CBD-induced lifespan extension [94]. It is noteworthy that these genes play a pivotal role in the intricate process of autophagy, influencing the formation of autophagic vesicles and the overall functionality of the autophagic machinery [107]. CBD also promoted longevity through the *sir-2.1*-dependent induction of autophagy (*sir-2.1* is an ortholog of sirtuin 1 (SIRT1) in *C. elegans*). SIRT1 regulates autophagy via modulation of the mammalian target of rapamycin (mTOR) and AMP-activated protein kinase (AMPK) activity [94].

Reactive oxygen species (ROS), including hydrogen peroxide (H_2_O_2_), hydroxyl radical (HO) and superoxide anions, can cause damage to cellular components, including DNA, proteins and lipids [108]. This damage can accumulate over time and contribute to the ageing process [109]. Several studies using *C.elegans* have demonstrated that the antioxidant properties of CBD contribute to its pro-longevity effects. One study investigated the glyoxalase pathway, an intrinsic antioxidant system, in the anti-ageing effects of CBD in worms [93]. The glyoxalase pathway involves glyoxalase enzymes which metabolise methylglyoxal (MG), a toxic metabolite that accumulates with age and may contribute to neurodegeneration [110]. MG treatment reduced the lifespan of various *C. elegans* strains, whereas treatment with 100 µM CBD attenuated the adverse effects of MG on lifespan. This finding was attributed to CBD enhancing the glyoxalase pathway via increasing the expression of glyoxalase enzymes. It was also found that CBD reduced the MG-induced increase in Aβ expression and oxidative stress in *C. elegans*.

CBD reduced ROS in N2 nematodes [97]. Another study examined the impact of CBD on ROS production in mitochondria and found that CBD exposure (1 µM) decreased the human Aβ peptide-induced increase in mitochondrial ROS in the transgenic GRU102 strain [111]. Further, CBD reduced elevated ROS production in the α-synuclein and 6-OHDA nematode models of PD. Moreover, CBD enhanced the expression of the antioxidant enzyme SOD-3 and extended the lifespan of nematodes [96]. CBDV, the propyl analogue of CBD, displayed antioxidant properties by prolonging survival in the juglone-induced oxidative stress model in various nematode strains [98]. In addition, CBDV upregulated the mRNA expression of *lys-7*, *mtl-1*, *sod-3*, *sod-4* and *sod-5*, which are antioxidative factors controlled by *daf-16*, a key transcription factor that regulates oxidative stress [98].

Structural analogues of CBD were shown to reduce ROS [97]. However, CBD-M1 (a CBD derivative with one phenolic hydroxyl group being methylated) and CBD-M2 (a CBD derivative with two methylated phenolic hydroxyl groups) were less effective than CBD itself in reducing ROS. Arguing for CBD’s antioxidant effect being due to direct scavenging of free radicals, CBD did not upregulate the expression of antioxidative genes such as catalase (CAT), superoxide dismutase (SOD) or glutathione S-transferase (GST). The synthetic analogues were also less effective than CBD in protecting against oxidative stress as indexed by juglone-induced mortality in *C. elegans* [97]. An interesting study administered medicinal cannabis oils to N2 worms and found antioxidant effects through a reduction in ROS, although these compounds contained a mixture of CBD and THC, and likely other compounds [112]. They did not test individual compounds alone which would have helped to deconvolute the effects observed.

In conclusion, CBD has the potential to increase the lifespan of *C. elegans* by promoting autophagy, decreasing oxidative stress and regulating the glyoxalase pathway. This effect is observed in both wild-type and transgenic strains, which are used to simulate neurodegenerative disorders such as AD and PD.

## Exogenous plant cannabinoid effects on healthspan in *C. elegans*

Healthspan refers to the period of an organism’s life during which it maintains good health and functional abilities, free from age-related diseases and disorders. In short, it refers to the number of “healthy life years” [113]. In ageing research, healthspan is considered to be a more relevant and useful measure than lifespan alone, as it allows researchers to assess the impact of various interventions on the overall health and function of the organism, rather than just focusing on its lifespan. There is no point to living longer without corresponding improvements in healthy function and well-being. In *C. elegans*, healthspan can be assessed by measuring various parameters such as mobility, muscle function/integrity, cellular accumulation of autofluorescent pigments that reflect ageing, and resistance to stress and disease. Many studies have shown that CBD diminishes age-related decline in physiological and behavioural function in *C.elegans*.

As *C. elegans* age, they exhibit decreased locomotion due to a decline in the function of the organism’s nervous system [70], as well as a decline in muscle function [114] and other physiological processes [115]. With age, the frequency of body movements, specifically body bends, decreases in *C. elegans*. CBD exposure (1 µM) increased the frequency of body bends over 11 days, with a significant increase observed on day 7 [94]. Exposing *C. elegans* to a CBD-rich cannabis extract (MGC1013) enhanced movement patterns [116]. Despite the presence of CBD in MGC1013, the increased motility observed could not be solely attributed to CBD concentrations in the extract, as treatment with extracts derived from other CBD-rich varieties (MGC1074 and MGC1122) did not affect body bends. Furthermore, treatment with pure CBD at the same concentration as found in MGC1013 did not produce any effect [116]. This highlights that cannabis constituents beyond CBD might be explored for their beneficial effects on healthspan.

The age-related diminution of locomotor activity is accelerated in *C. elegans* models of neurodegenerative disease [117]. To evaluate the impact of CBD on healthspan in a disease context, a transgenic strain that expressed human Aβ_1-42_ in muscle tissues (CL4176) was used as an *in vivo* model for AD [97]. In the study, CBD delayed the paralysis of CL4176 worms in a concentration-dependent manner within a range of 10–100 µM. Notably, treatment with 100 µM CBD robustly increased body bends [97]. In another study, CBD-dominant medicinal cannabis extracts increased body bend rate compared to controls in the GMC101 nematode line where Aβ is expressed in body wall muscle [112]. This effect was also observed in heat-stressed GMC101 nematodes [112]. Further, CBD 5 µM restored exploratory behaviour deficits in pan-neuronal Aβ_1-42_ expressing *C. elegans* [95]. CBD (25, 50 and 100 µM) concentration-dependently improved deficits in food-sensing and locomotory rate in the 6-OHDA model of PD in *C. elegans* [96]. Further, CBDV (50 µM) mitigated the impaired body bending observed in another PD model strain (NL5901) that expresses human α-synuclein protein in body wall muscle [98]. Notably, CBDV did not delay the onset of paralysis in an AD nematode model at the same concentration.

Pharyngeal pumping is a well-characterized feeding behaviour in *C. elegans* and is considered a healthspan indicator that reflects the organism’s ability to ingest and process nutrients [118]. The pharyngeal pumping rate declines with age in *C. elegans* [119]. Studies have demonstrated that CBD increased the pumping rate in normal ageing and age-related diseases. In normal ageing, 1 µM CBD increased the pharyngeal pumping rate on days 3 and 5 of repeated daily treatment [94]. Similar results were observed when testing CBD-dominant cannabis extracts in the N2 worm strain [112]. In CBD-rich varieties, only one chemotype (MGC 1013) increased the pumping rate in a dose-dependent manner [116]. It is noteworthy that a polar fraction of the extract was responsible for the effects, suggesting a role for other non-cannabinoid constituents, as the cannabinoids are non-polar [116]. The effects of CBD on pharyngeal pumping rate have also been examined in AD models. Treatment with both low (1 µM and 5 µM) [95, 111] and high (100 µM) [97] CBD concentrations enhanced pumping rate in models where Aβ was overexpressed in muscle and neurons. In a PD model that overexpresses α-synuclein in muscle (NL5901), CBDV 50 µM diminished the age-related decline in pumping rate [98]. The reproductive system of *C. elegans* shows a decrease during ageing [120]. Various approaches have been discovered that slow reproductive ageing, such as genetic mutations [121], small molecules [122] and environmental factors [123]. CBD exposure (1 µM and 5 µM) increased the number of laid eggs and increased the number of progeny (brood size) compared to the control group in both wild-type and Aβ transgenic worms [94, 95].

There is emerging evidence that CBD has neuroprotective effects in *C. elegans*. The normal ageing of neurons in *C. elegans* involves subtle morphological changes with no apparent neuron loss or axon deficit [124]. These morphological changes in neurons include irregularly shaped somas, disorganized microtubule arrangements and the occurrence of bubble-like lesions, beading and blebs resulting in the distortion of the neuron structure [69, 124]. These changes have been found in touch receptor neurons (TRNs), as well as dopaminergic neurons [69, 124]. A recent study investigated the impact of CBD on the normal ageing of both anterior (ALM) and posterior (PLM) TRN neurons in a transgenic strain expressing a TRN-specific mec-4p::GFP [94]. CBD treatment reduced the number of irregularly shaped somas of ALM neurons in both young and old worms. However, CBD only reduced the proportion of defective processes (branching, beading and blebbing) in PLM neurons in aged worms but not young worms. In a disease context, CBD derivatives with methylated phenolic hydroxyl groups (CBD-M1 and CBD-M2) mitigated Aβ-induced neurotoxicity, showcasing CBD’s remarkable neuroprotective effects [95, 97]. Further, CBD also protected dopaminergic neurons from reserpine-induced degeneration and locomotor deficits, which were associated with reductions in ROS and α-syn accumulation [125].

Studies have also addressed the impact of cannabis and cannabinoids on sensory and neurobehavioural function. A study found that cannabis extracts had varying effects on sensory responses and memory in worms [116]. The cannabis extracts reduced the response to a noxious odour, which if anything suggests a reduction in sensory function. However, a CBD-rich variety extract also reduced adverse memory responses in nematodes [116]. Neuronal Aβ expression induces toxicity, leading *C. elegans* to exhibit deficiencies in their chemotaxis behavior [126]. CBD at both low (5 µM) and high (100 µM) concentrations increased chemotaxis indexes in Aβ overexpressed strains [95, 97].

## Anti-ageing effects of cannabinoids in fruit fly, zebrafish and mice

**Fig. 6:**
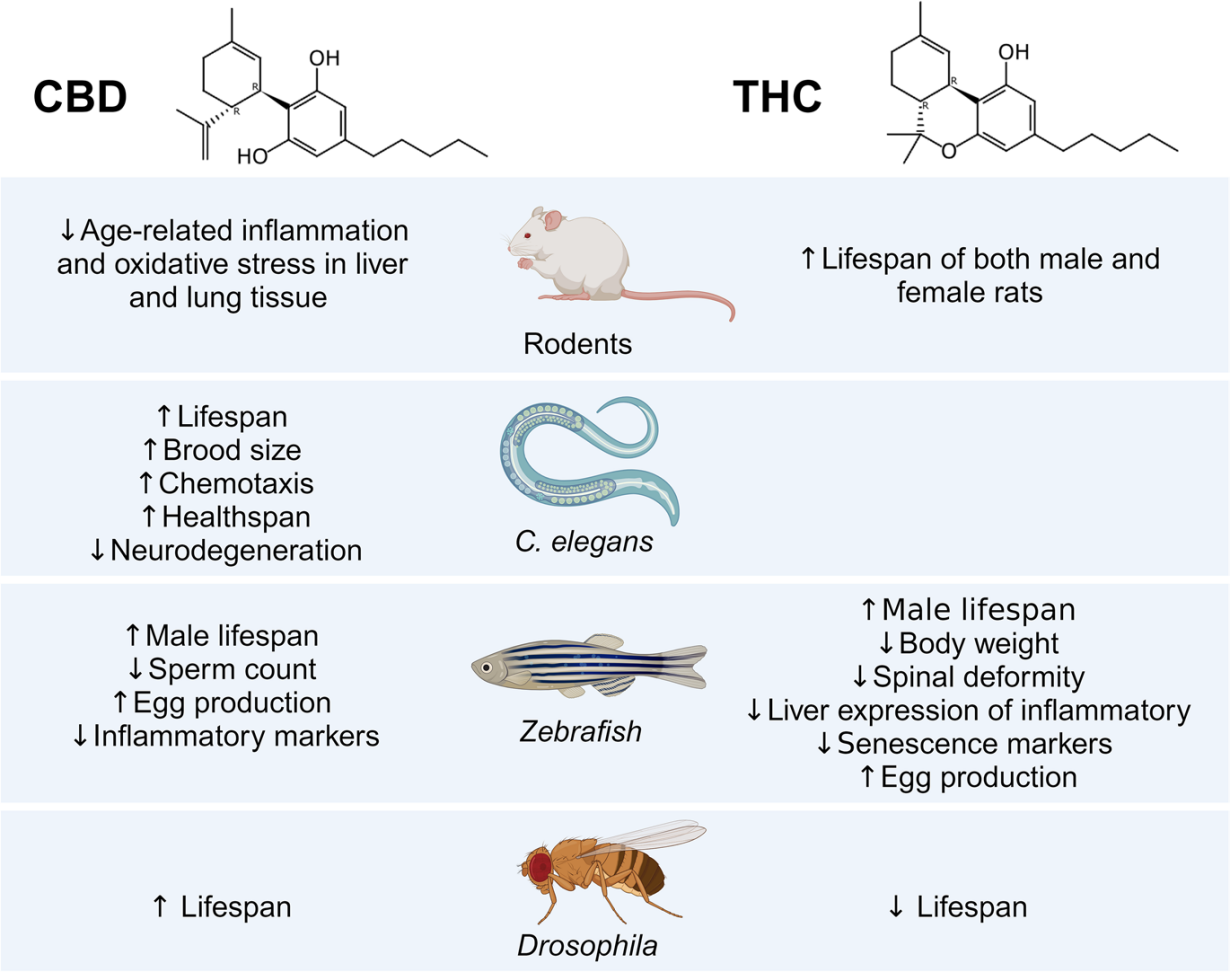
An overview of the anti-aging effects of CBD and THC in rodents, zebrafish, *C. elegans* and *Drosophila*

A recent study assessed the impact of cannabinoids on ageing in *Drosophila*. Flies treated with 3 µM CBD had significantly increased lifespan, whereas THC at the same concentration resulted in a modest decrease in longevity [127] (Fig. 6). Although CBD or THC at various concentrations had little effect on sleep and circadian-based behaviors, and the age-dependent decline in locomotor activity. Cannabinoids have been assessed for their effects on lifespan and healthspan in zebrafish (Fig. 6). THC increased survival of aged (30 months of age) male zebrafish at a low concentration (80 nM) but not at higher concentrations (400 nM and 2 µM) [128]. The low concentration of THC reduced body weight, spinal deformity and the liver expression of inflammatory and senescence markers (p16^ink4ab^, TNF-α, IL-1β, IL-6, PPARα and PPARγ). Moreover, it increased egg production, while the higher THC concentrations impaired fecundity. Another study showed that exposure to CBD (0.02, 0.1, 0.5 μM) during the F0 generation in zebrafish led to a remarkable increase in survival. Concentrations of 100 and 500 nM CBD increased survival in male zebrafish, but not female zebrafish. Although there was a strong non-significant trend of increased survival in the females with as great as a 50% increase in survival at 100 nM; it is possible that the study was underpowered. Although the 500 nM CBD concentration reduced sperm count in the males, 100 nM CBD increased egg production in the female fish. Additionally, CBD reduced the weight and length of zebrafish, although CBD reduced age-related increases in the inflammatory markers TNF-α and IL-1β in males and females. Unlike THC, there was no effect on the senescence-associated markers p16 or IL-6.

**Fig. 7:**
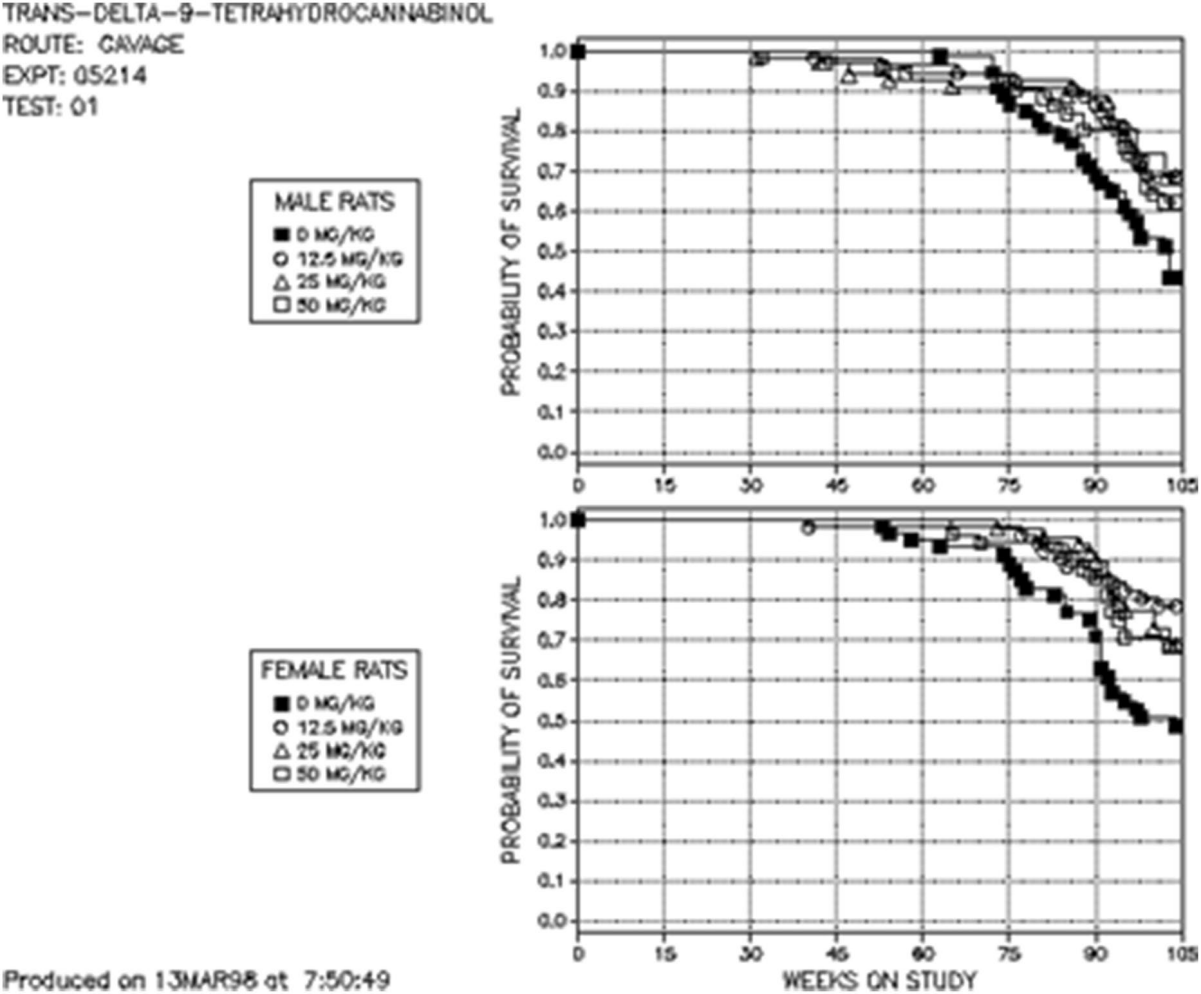
Rats administered THC by oral gavage for 2 years displayed significantly longer lifespan than vehicle controls. Adapted from the US National Toxicology Program [129, 130]

The available data on the effect of cannabinoids in rodent longevity is very limited. The study that assessed the impact of THC on rodent lifespan was conducted by the US National Toxicology Program (NTP) [129, 130]. In this study, mice and rats were dosed with relatively high oral doses of THC (12.5 mg/kg, 25 mg/kg and 50 mg/kg) over 2 years. THC considerably increased the lifespan of both male and female rats (Fig. 7). Although, no survival benefit of THC was observed in the mouse study. To the best of our knowledge, the effect of CBD on lifespan in rodents has not been examined. Although, there is evidence that CBD might improve healthspan in rodents. For example, one interesting study conducted on “middle-aged” rats (15 months old) showed that long-term treatment with CBD had beneficial effects on lung and liver health by lowering age-related oxidative stress and pro-apoptotic and inflammatory markers [131] (Fig. 6). Repeated CBD administration also had beneficial effects on emotional function and brain health in middle-aged female rats, where CBD reduced social isolation-induced behavioural despair and loss of brain-derived neurotrophic factor (BDNF) in the ventral striatum [132]. Consistent with this finding, repeated CBD treatment reduced behaviour despair in the forced-swim test in “older” rats (19–21 months of age) [133]. In addition, repeated CBD treatment improved blood glucose concentrations, nullified memory impairment and normalised neurodegeneration and neuroinflammation in response to chronic cerebral hypofusion in middle-aged diabetic rats [134]. It should also be pointed out that CBD and THC exposure promoted cognitive enhancing effects and improved brain function in mouse models of Alzheimer’s disease (for details see excellent reviews on this topic [135–137]).

## The effects of cannabinoids on mechanisms of ageing

There is an intense research effort in geroscience devoted to characterising the cellular and molecular mechanisms of ageing. The hallmarks of ageing include stem cell exhaustion, loss of proteostasis, mitochondrial dysfunction, genome instability, telomere attrition, epigenetic changes and cellular senescence [138]. Future studies are needed to assess whether cannabinoids can revert the hallmarks of ageing. We are beginning to see research in this area, with recent studies assessing whether cannabinoids affect cellular senescence. Preclinical research has shown that genetic and pharmacological deletion of senescent cells can extend lifespan and healthspan in animal studies [138]. Senescent cells lose the ability to divide due to telomere attrition and release mediators associated with the senescence-associated secretory phenotype (SASP) which promotes inflammation and cell damage in neighbouring healthy tissues. Thus, there is interest in developing agents that have senolytic (the ability to kill senescence cells) or senomorphic (the ability to diminish the SASP) activity.

In one study, both THC and CBD were examined in healthy and stress-induced premature senescent skin fibroblasts yielding perplexing results, with the title of the paper stating the cannabinoids prevented cellular senescence, but the balance of the data in the paper showing that the cannabinoids increased the viability of senescent cells [139]. Under conditions of replicative senescence, a cellular model of healthy ageing, treatment with THC (0.25–10 µM) non-dose-dependently increased senescence-associated β-galactosidase (SA-β-gal) staining following repeated, daily 2 h incubations for 2 and 15 days. Interestingly, CBD also non-dose dependently increased replicative senescent cell number as gauged by SA-β-gal after 2 days of treatment, although following 15 days of treatment CBD 10 µM reduced SA-β-gal activity. The authors themselves questioned whether this was a beneficial effect, and stated it was likely explained by CBD having overall cytotoxic effects on the cells, thus non-specifically decreasing all cell populations. CBD and THC also increased the viability of the skin fibroblasts that were induced to senescence with hydrogen peroxide; again, this might suggest that the cannabinoids increase the viability of senescent cells. However, 5 days of treatment with 2 µM CBD and THC reduced SA-β-gal in hydrogen peroxide-induced senescent cells. CBD and THC also enhanced wound healing of healthy and senescent skin fibroblast cells in the scratch assay. Notably, THC and CBD showed remarkable effectiveness in enhancing wound healing when compared to well-known anti-ageing nutrient signaling regulators like metformin, rapamycin and triacetylresveratrol.

In a follow-up study by the same lab, it was shown that the combination of CBD with triacetylresveratrol yielded significant benefits in senescent fibroblasts, by increasing viability and improving wound healing [140]. Notably, these studies also presented data highlighting that THC and CBD might have sirtuin-activating effects. SIRTs are signaling proteins that, among many functions, repair age-related DNA damage, and their activation has been suggested to extend lifespan and healthspan in model systems, albeit with some controversy [141, 142]. THC increased SIRT6 and CBD, when combined with triacetylresveratrol increased SIRT1 mRNA in skin fibroblasts [139, 140].

CBD treatment triggered cellular senescence in primary human Sertoli cells, a condition associated with male reproductive toxicity [143]. This effect was evident as CBD enhanced the expression of SA-β-gal and upregulated a set of genes related to SASP, along with activating the p53 signaling pathway [143]. In human astrocytes, CBD protected against Aβ-induced mitochondrial ROS and cellular senescence as gauged by SA-β-gal staining and senescence markers p16, p21 and p53 [111]. These protective effects of CBD were attributed to the activation of Parkin-dependent mitophagy which is autophagy specific to the removal of damaged mitochondria. In conclusion, these results highlight that cannabinoids do influence cellular senescence, but more research is needed to further illuminate the nature of the relationship. It would also be of interest to assess the effects of the cannabinoids on other hallmarks of ageing.

## Conclusion

Ageing is a complex and multifactorial process that occurs as a gradual accumulation of cellular damage in various tissues of the body, leading to a decline in physiological functions across all systems. One main aim of ageing research is to identify compounds that can postpone deteriorative changes linked to ageing. Finding interventions that promote healthy ageing may provide a paradigm shift in medicine, by targeting the common denominator of many diseases, that is, the ageing process. With the ongoing trend of cannabis legalisation globally, there is a demand for research that explores the impact of cannabis and cannabinoids on healthy ageing and diseases of ageing. The current review highlights that cannabinoids, whether endogenous or exogenous, extend lifespan and healthspan in model systems. However, more research is needed to observe whether these results translate in mammalian systems and ultimately in the clinic. The anti-ageing effects of cannabinoids have a number of different mechanisms, including the reduction of oxidative stress and the triggering of autophagy. More research is needed to further explore the anti-ageing mechanisms of the cannabinoids and to more comprehensively examine their impact on the hallmarks of ageing including cellular senescence. The development of anti-ageing agents that tone up endocannabinoid transmission could also be examined. Moreover, plant cannabinoids beyond CBD could be explored, as well as other cannabis constituents, alone and in combination. In addition, given the robust findings with CBD, chemical analogues of CBD might be developed. The current review underscores that there is much promise for the further development of cannabinoids as anti-ageing agents, to improve healthy ageing and general well-being.

## Data Availability

All the data is available on the request of the authors.
